# Medicinal Properties and Active Constituents of* Dracocephalum kotschyi* and Its Significance in Iran: A Systematic Review

**DOI:** 10.1155/2019/9465309

**Published:** 2019-05-06

**Authors:** Parisa Heydari, Maryam Yavari, Peyman Adibi, Gholamreza Asghari, Syed-Mustafa Ghanadian, Gabriel O. Dida, Faham Khamesipour

**Affiliations:** ^1^Department of Persian Medicine, Faculty of Medicine, Isfahan University of Medical Sciences, Isfahan, Iran; ^2^Department of Traditional Medicine, Isfahan University of Medical Sciences, Isfahan, Iran; ^3^Integrative Functional Gastroenterology Research Center, Isfahan University of Medical Sciences, Isfahan, Iran; ^4^Department of Pharmacognosy, School of Pharmacy and Pharmaceutical Sciences, Isfahan University of Medical Sciences, Isfahan, Iran; ^5^Department of Pharmacognosy, Faculty of Pharmacy, Isfahan University of Medical Sciences, Isfahan, Iran; ^6^School of Public Health and Community Development, Maseno University, Kenya; ^7^Department of Community and Public Health, Technical University of Kenya, Nairobi, Kenya; ^8^Cellular and Molecular Research Center, Sabzevar University of Medical Sciences, Sabzevar, Iran; ^9^Shahid Beheshti University of Medical Sciences, Tehran, Iran

## Abstract

**Objectives:**

* Dracocephalum* has over 60 species and is found mainly in the temperate regions of Asia and Europe. One of these species,* i.e., Dracocephalum kotschyi *Boiss, is known to have a number of medicinal properties and active ingredients in many parts of the world. Despite being an endemic wild-flowering plant of great importance, the plant is currently considered endangered in Iran. Besides, there is paucity of information on the significance of the medicinal properties and active constituents of* D. kotschyi* among the Iranian people. On that account a systematic review of studies reporting on the medicinal properties and active ingredients and its significance to human and animal health was conducted and the existing knowledge gaps were identified.

**Methods:**

The Preferred Reporting Items for Systematic Reviews and Meta-Analyses (PRISMA) guidelines were used in the search for published articles on medicinal properties and active ingredients of* D. kotschyi* and its significance on humans and animals in Iran. The search was confined to scientific articles from repositories of popular data bases and search engines among them PubMed, Web of Science, Google Scholar, Science Direct, SpringerLink, and Scopus. The search narrowed down on scientific journals, books, and book chapters focusing on the medicinal properties of* D. kotschyi* in Iran for the period between 1970 and 2018.

**Results:**

A total of 1158 scientific articles were sourced from the various databases, out of which 38 met the search criteria and qualified for this review. The studies were conducted in only 9 of the 31 provinces of Iran, with a large proportion in Isfahan province, central Iran. The studies showed that all plant parts (roots, aerial parts, flowers, and leaves) had active constituents. Essential oils and aerial plant parts were the main components studied. Nevertheless, the most frequently reported constituents were xanthomicrol, limonene, luteolin, geranial, apigenin, and calycopterin. A number of medicinal properties were reported among them antioxidant, antibacterial, anticancerous, antinociceptive, antihyperlipidemic, antispasmodic, cytotoxic, and immunomodulatory effects. The plant was also reported to be a remedy for inflammatory pain, headaches, congestion, liver disorders, ulcer, fever, renal pain, dyspepsia, stomach ache, abdominal pain, joints pains, muscle spasm, congestion, bloating, and wound healing effects, among others.

**Conclusion:**

This review has shown that* D. kotschyi *is an important medicinal plant with a large number of active constituents and great potential to safeguard human and animal health in Iran. However, over utilization of the* D. kotschyi* plant is already endangering its existence. Nevertheless, more studies need be conducted across the country.

## 1. Introduction

Plant products have been used in diseases prevention and treatment of disorders for decades [1]. According to Kinghorn* et al.* (2011) [2] and Newman and Cragg (2012) [3], numerous pharmacologically active drugs have been derived from natural resources including medicinal plants. The therapeutical role of a number of plants in diseases management is still being researched and used. The little side effects associated with the use of most medicinal plants coupled with their ease of availability and affordability make the use of medicinal plants popular among populations [4].

**Figure 1:**
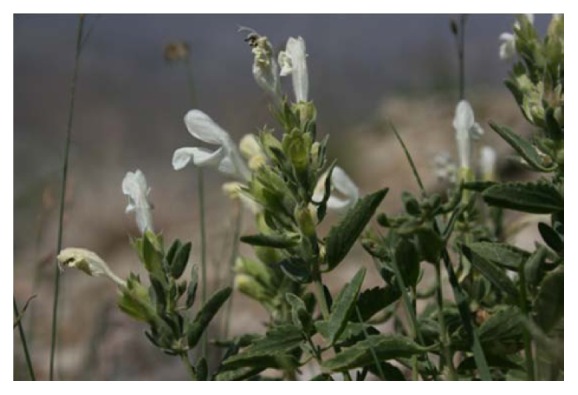
*Dracocephalum kotschyi* (picture courtesy of: http://www.gloria.ac.at/?l=430).

*Dracocephalum *is a genus of about 60 to 70 species of flowering plants in the family Lamiaceae, native to temperate regions of the Northern Hemisphere [5, 6]. These flowering plants, collectively called dragonhead, are annual or perennial herbaceous plants or subshrubs, growing to between 15 and 90 centimeters tall and can be prostrate or erect [7]. Their stems are square and bear simple leaves arranged oppositely or in whorls, while the plant is also characterized by tubular two-lipped flowers lobed at the base and the upper lip.* D. kotschyi *Boiss is among the most important* Dracocephalum* species. Morphologically,* D. kotschyi *is a short perennial herbaceous plant that is woody below, with stems measuring between 10 and 20 cm long and covered (Figure 1).

The plant has small pubescent leaves that are calyx two-lipped, with upper lip three-toothed, stamens, and flowers in verticillaster in the upper leaves [7, 8].* Dracocephalum kotschyi *is enriched in various constituents of essential oils including citral, caryophyllene, terpinyl, acetate, limonene, *α*-terpineol, *δ*-3-carene, *α*-pinene, terpinen-4-ol, geranial, limonene-10-al, 1,1-dimethoxydecane, Gerania, *α*-pinene [9], and flavonoids such as calycopterin, xanthomicrol, isokaempferide, luteolin, apigenin, luteolin 7-O-*β*-D-glucopyranoside, luteolin 3′-O-*β*-D-glucuronide, apigenin 4′-O-*β*-D-glucopyranoside, acacetin 7-O-*β*-D-glucopyranoside [10–14], limonene-10-al, and limonene, among others [15].

Flavonoids include over 4000 structurally related compounds existing in nature either as free aglycones or glycosidic conjugates and are generally classified according to their chemical structures into flavones, flavanones, flavanols, flavonols, and anthocyanidins [16]. These flavonoids appear to play important roles in the biological activities while the difference in antioxidant activity of extracts may be attributed to the difference in the total phenolic and flavonoid contents [14]. The antioxidant activity is generally attributed to phenolic compounds in plant extracts [17].

*Dracocephalum kotschyi* plant has been used widely as part of modern medicine for the treatment of many infectious diseases, as well as in the prevention of tumor proliferation across the world [18]. Several biological and pharmacological properties of* D. kotschyi* have been reported including antibacterial [19], antifungal, and anti-inflammatory [20].* D. kotschyi *has a number of medicinal properties among them antispasmodic, analgesic, antihyperlipidemic, and immunomodulatory activities [21, 22]. Flavonoids have antibacterial properties [23], while limonene and *α*-terpineol are responsible for antinociceptive properties of the essential oil of* D. kotschyi.* Methoxylated flavones such as apigenin, luteolin, isokaempferid, crisimaritin, penduletin, and xanthomicrol are responsible for the anticancer effects [24, 25], while phenolic compounds such as caffeic acid, chlorogenic acid, phenylpropanoids, and flavonoids are probably responsible for the antioxidant activity [26].

A study by Fattahi* et al.* (2013) [24] on hairy root lines induced by infection with* Agrobacterium rhizogenes* LBA 9402 was confirmed by PCR detection of rolC and aux1 genes and their capacity to grow and biosynthesize rosmarinic acid and surface flavonoids. Two types of morphology, typical hairy root and callus-like, were observed in the induced root lines. The rolC and aux1 genes were detected in the genome of both morphological types of root lines, although aux1 was more frequently observed in callus-like roots, indicating the capacity of the obtained hairy root lines to produce rosmarinic acid and methoxylated flavonoids. Rosmarinic acid content in hairy root lines ranged from 10 to 1500 mu g/g DW, which at its peak was 15 times higher than in the intact control roots. Surface flavonoids were identified in most hairy root lines, some of which showed a surface flavonoid content higher than the roots of the whole plant but generally lower than the plant leaves [23].

The* in vitro* cytotoxic, antiproliferative, and apoptotic effect of this plant against lung cancer cell lines was determined [27]. The morphological changes in cells were identified, with the most effective fractions being CH_2_Cl_2_ fraction, essential oil (EO), and luteolin [28]. Luteolin has multiple biological properties such as anti-inflammatory, antioxidant, and anticancer activities [28, 29]. The effect of flavonoids on inhibition of tumor cells has been reported for* D. kotschyi *with flavonoids of this plant being regarded as the most effective chemicals [30]. Studies by Sarvestani et al. [31] investigated the effect of a natural neuroprotective flavonoid, calycopterin, on H_2_O_2_-induced disruption of phase II detoxifying enzyme system and cAMP response element binding protein (CREB) phosphorylation and found that H_2_O_2_ decreased mitochondrial membrane potential (MMP), while calycopterin prevented this decrease in MMP in presence of H_2_O_2_. Luteolin can induce apoptosis in tumor cells such as epidermoid carcinoma, pancreatic tumor, leukemia, and lung cancer [24].

Farimani et al. [32] also investigated the neuroprotective potential of a natural flavonoid, calycopterin, against H_2_O_2_-induced cell death in differentiated pc12 cells and found that calycopterin not only protects pc12 cells against H_2_O_2_-induced apoptosis but also defends against the destructive effect of oxidative stress on the criteria of neural differentiation. Calycopterin decreased Endoplasmic Reticulum (ER) stress-associated proteins including calpain and caspase-12 and suppressed Extracellular Signal-Regulated Kinase (ERK), c-Jun N-terminal Protein Kinase (JNK), and p38 Mitogen Activated Protein Kinase (MAPK) phosphorylation, presenting a promising approach for the treatment of neurodegenerative diseases.

*D. kotschyi* has also been used in traditional medicine for stomach and liver disorders, headache, and congestion [33], as painkillers and for treatments of kidney complications, toothaches, and colds. They have also been reported to have antirheumatism, antitumor [31], antimutagens, antioxidant, antiseptic, and stimulant properties [32, 33], and antidiarrhoeal activities of hydroalcoholic and hexane extracts were observed in mice. The researchers suggested that the antispasmodic effect of apigenin, a flavonoid constituent, could be a suitable remedy for treatment of gastrointestinal disorders in which smooth muscle spasm and/or diarrhoea plays significant roles [34]. The leaf extract of* D. kotschyi* has various complex constituents which have been reported to have immunomodulatory [13, 21] and trypanocidal effects [11]. Research has also confirmed their roles as antiarthritic, antispasmodic, hypoglycemic, antigastric ulcer, antibacterial, and antitumor activities [34–36].

Iran with 1.64 million km^2^ areas has over 7500-8000 plant species, majority of which are medicinal [37]. Up to eight species of* Dracocephalum* including* D. kotschyi, D. aucheri, D. moldavica, D. multicaule, D. polychaetum, D. subcaitatum, D. surmandimum,* and* D. thymifolrum* have been reported in Iran [38]. These plant species have been used in traditional medicine for the treatment of aliment such as congestion, headache, stomach ache, liver diseases, among many others [38].* Dracocephalum kotschyi*, known locally as Badrandjboie-Dennaie and Zarrin-Giah, is endemic in Iran where it grows in high and mountainous areas [14]. The plant is mainly distributed in the northern and central parts of the country but is also widely grown and cultivated in various temperate regions [39]. However,* D. kotschyi *Boiss is among the plants considered as being endangered (EN) in Iran because of their low distribution, overuse, and increased consumptive demand from the population [40]. This review summarizes the role of* D. kotschyi* and its active ingredients in diseases prevention and treatment through the modulation of various biological pathways in Iran.

## 2. Materials and Methods

### 2.1. Review Design

In this systematic review, the Preferred Reporting Items for Systematic Reviews and Meta-Analyses (PRISMA system) was used to select publications reporting on* D. kotschyi* in Iran between and 1970 and 2018. Using the PRISMA guidelines, published literature of studies on the medicinal properties and active compounds of* D. kotschyi* conducted within Iran was systematically searched in PubMed, SpringerLink, Scopus, Web of Science, WHOLIS, and U.S. Center for Disease Control and Prevention (CDC) databases. To maximize the search and reduce selection bias, the search was restricted to English articles using key terms such as “Medicinal properties of* Dracocephalum*”, “Lamiaceae”, and “*D. kotschyi*” in Iran, among others. During the initial search, all articles identified from the indexed databases were first selected based on their titles and later screened for eligibility based on the content of their abstracts. A full-text review of all relevant articles was then conducted, using a checklist (S1). During the review, data was extracted from the selected studies by filling a table containing several subsections like: year of study, the active compound/ medicinal properties, study design, study region, and author. The extraction of information followed the PRISMA guidelines and a check list.

### 2.2. Inclusion and Exclusion Criteria

Only articles describing findings on* D. kotschyi *in Iran were included in this review. All nonverified sources of information and studies conducted outside Iran were excluded.

### 2.3. Study Article Selection

After assembling different publications from various sources, duplicates were excluded and titles and abstracts of the remaining articles evaluated critically. Those that did not address* D. kotschyi *and its significance to the Iranians were excluded. Full-text publications that were not written in English were also excluded just as those that were addressing other medicinal plants besides* D. kotschyi* in Iran. Risk of bias was reduced by inclusion of published reports only and exclusion of studies whose research methodology was not comprehensive.

## 3. Results

### 3.1. Articles Included in the Review and the Geographical Distribution

**Figure 2:**
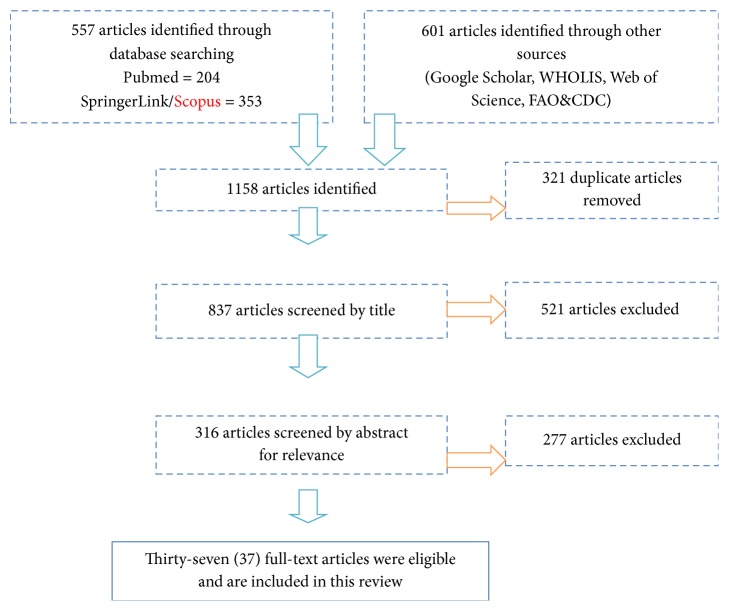
The PRISMA flowchart diagram describing the articles analysis process for inclusion in the systematic review (adapted and modified from Moher* et al*. (2009) [41].

The PRISMA flowchart shows the article selection criteria used to select articles for this review (Figure 2) [41]. A total of 557 articles were sourced from indexed scientific databases (PubMed, SpringerLink, and Scopus), and another 601 were sourced from generalized searches in Google Scholar, Web of Science, WHOLIS, FAO, and CDC databases to make a sum total of 1158 articles. All the articles were listed in MS Excel and 321 articles presenting duplicate titles removed to obtain 837 articles. Further screening was done by title and relevance and a total of 521 articles excluded from the review, leaving a subset of 316 articles. The 316 articles were further assessed for eligibility by reading through the abstract. A total of 277 articles were excluded at this stage following the subject matter leaving 37 articles as eligible for inclusion in this review.

Out of the initial 1158 articles sourced from the various databases, 38 met the search criteria and were included in this review. In the current review, studies on* D. kotschyi* were reported in only 9 (29%) of the 31 provinces of Iran, with most of them being carried out in Esfahan (32.5%). Others were reported in Tehran, Lorestan, Mazandaran, North Khorasan, west Azerbaijan, west Khorsan, Alborz, and Fars. This however leaves over 70% of the country's provinces. Ashrafi et al. [8] observed that* D. kotschyi *is native in Iran and grows wild in regions lying between 2000 and 3200m altitudes. However, in recent times,* D. kotschyi *is reported to be an endangered plant in Iran following excessive harvesting, limited distribution areas, and a lack of cultivation and domestication of the* D. kotschyi* plant [42].

## 4. Discussion

### 4.1. Usefulness of* D. kotschyi* as Traditional Medicine

The current review has shown that various parts of the plant (*D. kotschyi*) including roots, flowers, leaves, and even whole plants have been used by various researchers in the study of its active ingredient and medicinal properties. Consistent with the review findings, Mashayekhan* et al*. [43] concurs that the local people of Iran usually utilize every part of the plant. Nevertheless, essential oils and aerial plant parts were the main components of the* D. kotschyi* plant that were investigated. Studies of different parts of* D. kotschyi* plant show that aerial parts of* D. kotschyi* plants are sources of valuable flavonoids and essential oils [10, 20, 44, 45] while its seeds are rich in linolenic, oleic, and linoleic acids [46]. According to Mashayekhan* et al*. [43] and Mohammadi* et al*. [47], the most frequently used plant parts in the preparation of herbal remedies are leaves, fruits, seeds, flowers, bark, and gum [47]. According to Lee* et al*. [48], ease of availability, fewer side effects, and reduced toxicity of some aromatic medicinal plants make their application for treating infectious diseases in many parts of the world very ideal.

Saeidnia* et al*. [49] studied components of the oil of* D. kotschyi *collected from Iran and established that the oil contained geranial, limonene-10-al, limonene, and 1,1-dimethoxydecane [39], while Javidnia* et al*. [50] reported the main components of the oil of* D. kotschyi *to be *α*-pinene, caryophyllene oxide, terpinen-4-ol, and germacrene [49]. Golshani* et al*. [25] and Yaghmai and Tafazzoli [51] reported citral, myrcene, *β*-caryophyllene, and terpinyl acetate as the main constituents of* D. kotschyi *from northeast mountains. Yaghmai and Taffazoli [51] and Saeidinia* et al*. [49] also reported that the main components found in the essential oil were *α*-pinene, neral, geraniol, *α*-citral, limonene, cyclononadiene, terpinene-4-ol, linalool, carveol, myrcene, germacrene-D, isopinocarveol, and *α*-terpineol.

A number of compounds have been identified in the essential oil from* D. kotschyi* in Iran [44, 45, 49, 51]. Golparavar* et al*. [52] reported that the major components in the oil* D. kotschyi *were limonene, carvacrol, *γ*-terpinene, *α*-pinene, 2-methyl-1-octen-3-yne, camphene, myrcene, and *α*-terpinene [53]. Some of the constituents of alcoholic extract from* D. kotschyi* include calycopterin, xanthomicrol, isokaempferide, luteolin, apigenin, luteolin 7-O-beta-D-glucopyranoside, lutcolin 3′-O-beta-D-glucuronide, apigenin 4′-O-beta-D-glucopyranoside, acacetin 7-O-beta-D-glucopyranoside, and rosmarinic acid [10].

### 4.2. Active Ingredients of* Dracocephalum kotschyi *Boiss

A number of researchers have presented various constituents of* D. kotschyi*, with the most frequently reported being limonene, luteolin, geranial, apigenin, and calycopterin. A comparison of the main components of* D. kotschyi *from different provinces is presented in Table 1 [8, 10, 11, 14, 17, 18, 23–25, 31, 39, 44, 45, 48–66]. In spite of some similarities in reported components, there are significant quantitative and qualitative differences between the samples obtained from different locations in Iran. These differences might be due to different research methodologies involved, climatic, seasonal, and geographic conditions; harvest periods; distillation techniques, among others [52].

### 4.3. Medicinal Properties of* D. kotschyi* and Their Mode of Action

Pharmacological studies have confirmed some medicinal properties of* D. kotschyi* including antinociceptive [23], antihyperlipidemic [20], immunomodulatory [21], and cytotoxic [24] effects. The essential oil from* D. kotschyi* has strong spasmolytic activities on isolated ileum [67, 68]. Lipopolysaccharide- (LPS-) stimulated J774.1 mouse macrophages cultured in the presence of the plant extract and significantly reduced the expression of key mediators of inflammation [49]. Boiled extract of this species has been reported to be used as antispasmodic agent in Iranian traditional medicine [49].

### 4.4. Antioxidant Activity

Antioxidant activity of* D. kotschyi *has also been reported and is mainly due to chemical structures of compounds, which allow them to act as reducing agents [69]. The redox properties of phenolic compounds enable them to act as reducing agents, hydrogen donors, and singlet oxygen quenchers [70]. Solvent polarity plays an important role in extraction of phenolic compounds. In this regard, methanol was a better solvent in extraction of phenolic compounds [71]. The quantity of luteolin in* D. kotschyi *was found to be 1061.005 *μ*g/g of dried plant. The results of this investigation indicated that luteolin plays major role in the antioxidant activity of the plant. Previously, luteolin was also isolated from the methanol extract of* D. kotschyi* by Gohari* et al*. [10] and Fattahi* et al*. [14] who also confirmed its antioxidant activity [14].

A number of studies have also been done to evaluate antioxidant activity in different crude extracts of the leaves of* D. kotschyi. *Result of the current finding suggested that the methano-based crude extracts of* D. kotschyi *could be used as a natural antioxidant. Antioxidant activity of* D. kotschyi* is mostly due to flavonoids content such as luteolin, apigenin, cirsimaritin, xanthomicrol, and rosmarinic acid [61]. Among these, luteolin has been shown to have multiple properties such as anti-inflammatory, antioxidant, and anticancer activities [27, 28]. In one study, a GC-MS analysis of the essential oils identified 15 components as antioxidant, with (E)-*β*-ocimene as principal ingredient, whose percentage varied pursuant to the phonological stage (53.28 ± 0.7, 47.2 ± 0.7, and 33.0 ± 0.3 for vegetative, flowering, and fruiting, respectively). The other principal compounds identified were nerol at vegetative (36.38 ± 0.7), nerol/methyl geranate (15.5 ± 0.2 and 8.3 ± 0.1) at flowering, and *α*-pinene/geranial/geraniol (16.7 ± 0.2, 14.8 ± 0.2, and 11.5 ± 0.2) at fruiting stage [72].

However, other compounds such luteolin-7-O-glucoside, apigenin-7-O-glucoside (cosmosiin), luteolin 3′-O-*β*-d-glucuronide, luteolin, apigenin, cirsimaritin, isokaempferide, penduletin, xanthomicrol, calycopterin, and the polyphenol rosmarinic acidquercetin and apigenin also exhibited high level of antioxidant activity and appeared to play a major role in the biological activities [14]. Overall, the majority of the studies show that extract exhibiting highest amount of flavonoid and phenolic compounds (methanol extract) showed greatest antioxidant activity, with luteolin being the main contributor of antioxidant of this plant. Saeidnia* et al*. [49] in a study of* D. kotschyi *collected from Tochal Mountain, north of Tehran, Iran, showed that this plant had limonene, geranial, neral, b-sitosterol, oleanolic acid, ursolic acid,* p*-mentha-8-en-1,2-diol, colosolic acid, and limonene [11].

### 4.5. Anticancerous Activity of* D. kotschyi*

Cancer is multifactorial disease and major health problem worldwide. The alteration of molecular/genetic pathways plays role in the development and progression of cancer. The treatment module based on allopathic is effective on one side but also shows adverse effect on the normal cell. In contrast, studies showed that some methoxylated flavonoids from* D. kotschyi* such as xanthomicrol [24] and calycopterin [66, 73] have safe and great anticancer properties [24, 25, 29]. The leaf extract of* D. kotschyi* showed a higher cytotoxic effect against A172, A2780-s, HL60, KB, K562, MCF-7, Saos-2, Hela, A2780-cp, A549, A375, and HFFF-P16 cell lines [24], while Faham* et al*. [70] reported calycopterin inhibition of proliferation of lymphocyte in a dose-dependent manner (IC50 = 1.7 *μ*g/mL); Moghaddam* et al*. [27] isolated eight flavonoid aglycones (luteolin, naringenin, apigenin, isokaempferide, cirsimaritin, penduletin, xanthomicrol, and calycopterin) from the aerial parts of* D. kotschyi* and reported that they had anticancer activities.

In addition, Moghaddam* et al*. [27] in a study that sought to examine the cytotoxic effects of fractionated* D. kotschyi* extracts on Calu6 and Mehr-80 cancer cell lines and to find other compounds with significant anticancer properties in* D. kotschyi* established that methoxylated hydroxyflavones (cirsimaritin, penduletin, xanthomicrol, and calycopterin) showed selective activities against tumor cells. The researchers demonstrated that partially nonpolar fractions from* D. kotschyi* extract exerted potent cytotoxic effects on Calu-6 and Mehr-80 cells. The most effective fraction was the CH_2_Cl_2_ one followed by EtOAc fraction and then the methanolic extract. Furthermore, plant EO showed suitable cytotoxic effects. The water (aqueous) fraction did not exhibit any significant anticancer activity in any cell lines (IC50 > 200 *μ*g/mL). Luteolin, the major compound of the total extract exhibited significant anticancer activity. All the effective fractions had more potent inhibitory effects on Calu-6 cells compared to Mehr-80 cells. DNA fragmentation analysis and morphological changes in cells supported the data extracted from cytotoxicity identification in MTT assay. In many studies, essential oil components had shown dose-dependent antiproliferative effects on cancer cells, which make them potentially interesting for experimental cancer treatments.

### 4.6. Effects of* D. kotschyi* on Tumor Cells

*D. kotschyi* plays an important role in the regulation of apoptotic process. Moghaddam* et al*. [27] isolated eight flavonoid aglycones from the aerial parts of* D. kotschyi*: luteolin, naringenin, apigenin, isokaempferide, cirsimaritin, penduletin, xanthomicrol, and calycopterin. Of these, the methoxylated hydroxyflavones (cirsimaritin, penduletin, xanthomicrol, and calycopterin) showed selective activities against tumor cells. Also, through bioassay-guided fractionation and using HL-60 human promyelocytic leukemia cell line, a flavonoid identified as xanthomicrol was isolated as an antiproliferative constituent present in its extract with preferential activity towards malignant cells [24]. Another study by Moghaddam* et al*. [27] also reported that flavonoids from* D. kotschyi* exhibited preference for tumor cells. The study findings further revealed that luteolin induced apoptosis in tumor cells indicating that it has apoptosis inducing effects in the target organ. Thus, isolated compound and chief constituents from* D. kotschyi *show a range of activities affecting multiple targets and also play a role in the induction of apoptotic cell death in cancer, giving clue on the means of developing drugs' effectiveness against cancer in human [25, 29].

Graidist* et al*. [74] conducted a study in Thailand and found that four of the extracts (hexane, methanol, chloroform, and aqueous) assayed presented different cytotoxic activity against breast cancer line MDA-MB-231, in the in* vitro* screening. The four of different extracts may have antitumoral activity. Nevertheless, methanolic extract had the greatest activity; this value being within the concentration limit required for further purification flavonoids is known to be present in* D. kotschyi *[25, 29, 75].

The breast carcinoma is considered to be one of the most common malignant tumors. Therefore, the search for new drugs is imperative and the results of our investigation call for future isolation and characterization of the active constituents by bio-guided assay. In study of Moghadam* et al*. [27], luteolin was been shown to have effective antiproliferative properties against various cancer cell lines including human HL-60 leukemia [76], AGS human gastric adenocarcinoma [77], MDA-MB-435 and MCF-7 breast cancer, HT-29 colon carcinoma, DU-145 prostate cancer, SK-MEL5 melanoma, and DMS-114 lung cancer cell lines [78]. The observed antiproliferative activity of luteolin in this study was consistent with the previously reported results on AGS [77], HT-29 [78], and HL-60 [76, 78] cell lines.

Likewise, Jahaniani* et al*. [26] demonstrated that flavonoid of xanthomicrol was isolated from* D. kotschyi *and it had preferential antiproliferative activity against a number of cancerous cell lines compared with normal cells [24]. Researchers had reported the antiproliferative activity of naringenin and epigenin compounds against HT-29 [78] and HL-60 [76] cell lines. Xanthomicrol has been reported to exist in* D. kotschyi *together with a number of other flavonoid compounds [10, 66]; for only two of which,* i.e.,* apigenin and luteolin, significant data exist supporting their antiproliferative activities against various cancer cell lines [76, 79].

### 4.7. Effect of* D. kotschyi *on Angiogenesis

Angiogenesis is complex process that supplies blood to the tissue and essential for growth and metastasis of tumor [80]. Angiogenesis is regulated by activators as well as inhibitors. Medicinal plants and their ingredients play a role in prevention of tumor growth due to their antiangiogenic activity. Inhibition of angiogenesis could be useful in tumor treatment. Previous studies reported that certain natural products such as aqueous extract of shallot [63], soybean trypsin inhibitor [27], a peptide from shark cartilage [81], and green tea extract [82] could inhibit angiogenesis on HUVEC three-dimensional model. The suppression of any phases of angiogenesis inhibits the formation of new vessels thus influencing tumor growth and metastasis [83]. One group of growth factor receptors critically implicated in angiogenesis is vascular endothelial growth factor receptors (VEGFR-1, -2, and -3), a subfamily of receptor tyrosine kinases (RTKs) [84]. Development of VEGF-Rs antagonists, which inhibit these molecules interacting with their ligands, is a validated therapeutic strategy of anticancer treatment. However, in Iran the medicinal properties of most medicinal plant including* D. kotschyi* on angiogenesis are not yet documented.

### 4.8. Effect of* D. kotschyi *on Cell Oncogenesis

An oncogene has been shown to play a significant role in the development and progression of tumors. For instance, Amirifard* et al*. [85] performed an experiment involving the lymphovascular system, including lymph nodes in axillaries, and found out that hormone receptor with HER2-neu oncogene had direct correlation with tumor location, patient age, and histological characteristics. However, although HER2-neu oncogene had no significant relationship, there was significant correlation between HER2 with ER and PR and also HER2 with ER, PR negative. The conclusion was that HER2-neu may be good prognostic and also predictive factor. Although such relationship has been demonstrated, the existence of -*κ*B, Bax and Bcl-2 factors in* D. kotschyi* needs investigation as the involvement of nuclear factor-*κ*B, Bax, and Bcl-2 in induction of cell cycle arrest and apoptosis by apigenin in human prostate carcinoma cells. Oncogene was previously observed [86].

### 4.9. Effect of* D. kotschyi* on PI3K/Akt Pathways

PI3K/Akt pathways show pivotal effect in the promotion of tumor. However, inhibition of PI3K/Akt pathways is one of the important steps towards regulation of tumor development. The phosphorylation of Akt is related to protection of cells from apoptosis [87]. Esmaeili* et al*. [73] demonstrated how calycopterin purified from* D. kotschyi* Boiss markedly dephosphorylated PI3K/Akt at 10, 25, 50, and 100*μ*M increased the phosphorylation of ERK1/2 phosphorylation of JNK and p-38 MAPK significantly increased at 50 and 100 *μ*M. These data suggest that calycopterin modulated the PI3K/Akt and MAPKs pathways in HepG2 cells [73]. Esmaeili et al. [73] further unravelled the possible roles and effects of PI3K/Akt and MAPKs in calycopterin-induced apoptosis by examining the changes in cell cycle and protein level due to G2/M cell cycle arrest and apoptosis in the presence or absence of specific inhibitors for PI3K (LY294002), ERK1/2 (U0126), JNK (SP600125), p38-MAPK (SB203580), and caspases (z-VAD-fmk) [73]. These results were consistent with those of Lee et al. that reported Akt, JNK, and p38 MAPK pathway to be directly related to the induction of apoptosis and the ERK1/2 pathway to be involved in the G2/M cell cycle arrest mechanism caused by calycopterin in HepG2 cells [88].

A study by Moghaddam* et al*. [27] evaluated the molecular mechanisms involved in the induction of apoptosis and antiproliferative activity exerted by leaf extract of* D. kotschyi* plant obtained from a herbal drug store in Isfahan province, Iran, and demonstrated that a flavonoid (xanthomicrol) contributed to its preferential antiproliferative activity against malignant cells. Further, the results showed that extract treated cells significantly decreased the protein expression such as IGF signalling molecules IGF-1R, Ras, Raf, p-Erk, and p-. Calycopterin triggers ROS and NO production, which induces mitochondrial oxidative/nitrative stress and inhibits the PI3K/Akt pathway and the subsequent release of cytochrome c followed by caspase-3 activation and apoptosis [25, 29].

### 4.10. Effect of* D. kotschyi* on NF-*κ*B Factor

The inhibitory effect of* D. kotschyi* extract on the lectin-induced cellular immune response was previously reported by Karrer and Venkataraman [89]. The production of TNF-*α* is important for the induction of NO synthesis in LPS-stimulated macrophages [90]. Among the extracts,* D. kotschyi* was shown to decrease production of TNF-*α*, although effect was not significant. However, in a different study the extract decreased production of IL-1*β*, which is known to play a crucial role in inflammatory response, a biologic function very similar to TNF-*α*, and involved in the pathogenesis of inflammatory diseases [91]. Inhibition of TNF-*α* and IL-1*β* secretion from macrophages by this extracts indicate their capacity to diminish immune reactions and provide further evidence that these plants may have potent immunomodulatory properties. In a study by Amirghofran* et al*. [57] investigating NO production and activity by lipopolysaccharide-stimulated mouse macrophages,* D. kotschyi*, extracts decreased secretion of IL-1*β* from the cells.

### 4.11. *D. kotschyi* as Anti-Inflammatory

Plants or their isolated derivatives are in the practice to treat/act as anti-inflammatory agents. Sadraei* et al*. [49] used acetic acid induced colitis to test anticolitis activity of* D. kotschyi* extract and found that apigenin was effective to reduce various assessed parameters of experimental colitis. In* D. kotschyi*, the active apigenin was found in the form of various acylated derivatives and as apigenin-7-O-glucoside [49]. A similar finding showed immunomodulator, anti-inflammatory, and antipyretic activities of oil form* D. kotschyi *seeds [9]. Amirghofran* et al*. [57] and Kalantar* et al*. [92] investigated the NO modulatory activity of the extracts of* D. kotschyi *and observed a decrease in secretion of IL-1*β* from the cells, indicating the presence of anti-inflammatory and macrophage inhibitory substances in this plant and suggested that the plant can be an alternative for the treatment of a wide range inflammatory diseases.

### 4.12. Antidiabetic Activities

A few studies have appreciated the antidiabetic effects of* D. kotschyi*. Antioxidant therapy acts as a protection against oxidative stress. Herbal products can improve antioxidant status and therefore improve complications of diabetes. Recent studies have shown that plants containing many antioxidant agents like flavonoids exert a protection against beta cell impairment. Flavonoids, the most prominent plant antioxidants, are a large class of phenolic compounds, acting as free radical scavengers [93], with protective effect of rutin on paracetamol and CCL_4_-induced hepatotoxicity in rodents. Luteolin, a* D. kotschyi*-derived flavonoid, possesses direct antioxidant activity and has shown the highest degree of free radical scavenging activity. Various biological actions of luteolin are mediated by inhibiting oxidative stress [94]. Administration of luteolin in diabetes provides protection against diabetic nephropathy or delays its development. Regulation of postprandial hyperglycemia is important. In this regard, retardation of glucose absorption in small intestine is a therapeutic protocol to achieve this goal. *α*-Amylase is an enzyme that catalyzes starch breakdown to maltose and then glucose, which is absorbed in gut. Based on the researches available, hypoglycaemic medicinal plants may act through this mechanism.

### 4.13. Antimicrobial Properties of* D. kotschyi*

Antimicrobial properties of physiologically active principles in medicinal herbs have led to the exploitation of plants as traditional medicine for the treatment of various ailments [95, 96].* D. kotschyi* and its ingredients have been shown to play a role in the inhibition of growth of numerous bacterial agents [22]. In Iran,* D. kotschyi *essential oils were stronger against Gram-positive bacteria in contrast to Gram-negative bacteria, with strong inhibitors of Oxa-48 positive* K. pneumoniae *strains reported [95], and this was attributed mainly to the activity of ethyl acetate and methanol found in flavonoids [96].

The essential oils in* D. kotschyi *effectively inhibit the growth of all tested food-borne pathogenic bacteria. A number of researchers have studied the antimicrobial activity of* D. kotschyi* essential oils and have attributed their efficacy to the presence of mostly active compounds [97], such as geranial *α*-pinene [98], geraniol acetate, geraniol [99, 100], neral [101, 102], and limonene [35]. Moreover, the components such as trans-caryophyllene, germacrene-D, *δ*-cadinene, *β*-pinene, *β*-myrcene, and sabinene which were found in lower levels could also contribute to the antimicrobial activity of the oils [98, 103, 104]. In fact, the synergistic effects of the diversity between the major and minor constituents present in the essential oils should be taken into consideration in accounting for their biological activity.

Overall, in comparison with Gram-negative bacteria, the antimicrobial effects of* D. kotschyi *essential oils were stronger against Gram-positive bacteria which is consistent with a general observation derived from studies with essential oils from many other plant species [105–107]. This generally higher resistance of Gram-negative bacteria could be associated with their outer phospholipid membrane, which is almost impermeable towards lipophilic compounds [108]. Lack of this barrier in Gram-positive bacteria makes the direct contact of the hydrophobic components of the essential oils with the phospholipid bilayer of the cell membrane possible. Such direct contact results in either an increase of ion permeability and leakage of vital intracellular constituents or failure of bacterial enzyme systems [109].

### 4.14. Wound Healing Effect

Although few studies appreciate the effects of* D. kotschyi* on wound healing, these have not well been documented in Iran. However, preliminary results of different studies about medicinal properties of different species of* Dracocephalum* have shown antinociceptive effect of* D. kotschyi* Boiss [23].

### 4.15. Other Medicinal Properties

Additionally,* D. kotschyi* Boiss which contains flavonoids is used in various illnesses including removing renal pain, analgesic; tonic seizure, dyspepsia, stomach ache, abdominal pain, and joints pains [110]. In Iranian traditional medicine* D. kotschyi *has been used as a remedy for treatment of a number of ailments among them inflammatory pain, ulcer and fever [5–7]. This medicinal plant also is used for many aliments such as muscle spasm, congestion, bloating, and other gastrointestinal disorders.* D. kotschyi *has is also applied as a warm herbal medicine for rheumatoid diseases and stomach disorders treatment. Its effectiveness for headache, constipation, abdominal pain anorexia, cough, and heartburn, among other, has recently been reported [110]. Previously, its effects on congestion and liver disorders have been described as well [111]. Moreover, antihyperlipidemic [20], antiepimastigote [11], and antivisceral [23] effects are among the other properties reported for* D. kotschyi*. The herb is known as “Sama” in Lorestan locally and it is traditionally used in cooking meat and fish and dairy processing (Local People Interview) and there is a general belief that the use of this traditional herb could reduce the microbial load of the meat and dairy products.

Herbaceous remedies of this plant have also been used in the treatment of headaches, digestive, and liver disorders, as well as flavor for tea and yogurt [68]. Its decoction is also reportedly used for the management of fever, analgesic, and rheumatoid arthritis [43, 47]. The cytotoxic effects of xanthomicrol on malignant cells have been demonstrated [11, 25, 29]. Xanthomicrol induces a dose-dependent apoptotic cell death using fluorescence staining method [112–114], and such types of ingredients play a role in diseases management through modulation of various genetic pathways [115, 116]. Leaf extracts also showed a mean blood glucose decrease in diabetic patients [58].

## 5. Conclusion

This review has noted that* D. kotschyi* has several beneficial ingredients that can be exploited for drug development. Currently, surmounting evidence suggest that medicinal plants have a number of medicinal properties often with minimal side effects. Results of this review further showed a relatively strong cytotoxic effect of methanol extract of* D. kotschyi *aerial parts and the high content of phenols and flavonoids in these extracts. Based on these results,* D. kotschyi *can be a suitable choice for designing and manufacturing anticancer drugs. However, as is evident from this review, the medicinal properties of* D. kotschyi* on a number of diseases, both in human and in animals such as antiviral, antifungal*, a*ntinephrotoxicity, neuroprotective, immunomodulatory, and growth promoting activities, have not been well evaluated. Additionally, research based on safety and toxicity, especially on the appropriate LD_50_ values through laboratory tests, and clinical studies are needed. Though some herbal products of* D. kotschyi* have been prepared and used traditionally by the locals in parts of Iran, its potential has not been fully exploited for better utilization across Iran and around the worldwide. Given that* D. kotschyi* is an endangered species in Iran, there is need for concerted effort to conserve and protect the plant and therefore subvert its eventual elimination.

## Figures and Tables

**Table 1:** Main components in *D. kotschyi *from different studies and provinces.

No.	Province	Extract (area)	Method of identification	Major constituents	References
1	Aladagh mountains (3000 m) near Bojnord in North Khorasan	Essential oil (Flower)	GC‐MS.	Citral, Caryophyllene, terpinyl acetate, myrcene, and Menthone	[51]

2	Bojnord, North Khorasan,	Essential oil (Flower)	GC-MS	Limonene, verbenone, *α*-terpineol, perillyl alcohol and Caryophyllene	[23]

3	Suburb of Sari, Mazandaran province, North of Iran	Essential oil (Aerial part)	GC-MS	*δ* -3-carene, limonene, carvacrol, 1,8-cineole and carvone.	[52]

4	Muteh protected region, Isfahan province	Essential oil (Aerial part)	GC-MS	*α*-pinene, caryophyllene oxide, terpinen- 4-ol and germacrene D.	[50]

5	Tochal Mountain near to Tehran	Essential oil (Whole plant)	GC-MS	Geranial, limonene-10-al, limonene and 1,1-dimethoxydecane	[45]

6	Garin mountains (3200 m) near to Alshtar in the of province Lorestan	Essential oil (Aerial part)	GC-MS	Geranial, *α*-Pinene, Geraniol acetate, Geraniol, Neral and Limonene.	[8]

7	Fars province	Powder (flower)	GC-MS	Linolenic acid, oleic and Linoleic acids	[53]

8	Isfahan	Essential oil (Flower and roots)	GC-MS	Geraniol, geranyl acetate (trace) and neryl acetate.	[54]

9	Lorestan	Powder (Leaf)	GC-MS	Flavonoids, Tannins	[51]

10	North Khorasan Province	Aerial parts (Powder)	GC-MS	Luteolin	[55]

11	Isfahan and Tehran	Powder (Leaf)	GC-MS	Xanthomicrol	[56]

12	Isfahan	Aerial part (green extract)	Observation	Green extract	[49]

13	Mazandaran	Essential oil (Aerial parts)	GC-MS	Limonene, *α*-terpineol and *α*-pinene	[57]

14	Alborz	Whole plant	GC-MS	Flavonoids, calycopterin, anthomicrol, isokaempferide, luteol, apigenin, g), luteolin 7-O-*β*-D-glucopyranoside, phenolic, rosmarinic acid	[10]

15	Sari in Mazandaran	Essential oil (Aerial parts)	GC-MS	Limonene, carvacrol, *γ*-terpinene, *α* -pinene, 2-methyl-1-octen-3-yne, camphene, myrcene and *α* –terpinene.	[48]

16	Mazandaran	Essence of the shoots	GC-MS	Limonene, cyclohexene, *α*-pinene, Sylosyfon, trans-limonene oxide.	[59]

17	Kansar–Isfahan	Essential oil (Aerial part –Flower)	GC-MS	Limonene-10-al, limonene	[60]

18	Muteh protected region in Isfahan	Essential oil (Aerial parts of the plant)	GC-MS	*α* -pinene, caryophyllene oxide, terpinen- 4-ol and germacrene D.	[54]

19	Isfahan	Essential oils	GC-MS	Longibornyl and acetate.	[49]

20	Khorasan	(Aerial parts and roots)	HPLC and GC- MS	Luteolin, quercetin and apigenin, phenolic, Gallic acid	[62]

21	Khorasan	Essential oil (Aerial parts)	HPLC, MS and NMR	Geranial, limonene and 1, 1-dimethoxydecane.	[62]

22	Isfahan	Aerial parts	GC-MS	Flavonoid aglycones (luteolin, naringenin, apigenin, isokaempferide, cirsimaritin, penduletin, xanthomicrol and calycopterin).	[25]

23	Mountain, Alborz, north of Tehran	Dried whole plants	GC–MS	Terpenoids and a phytosterol (1—8) [limonen-10-al, geranial, neral, b -sitosterol, oleanolic acid, ursolic acid, p-mentha-8-en-1,2-diol, colosolic acid, limonen-10-ol 10-O-b -D-glucopyranoside, and limonen-10-ol 10-O-b -D-glucopyranosyl-(1Æ2)-b –D glucopyranoside).	[11]

24	Tehran	Aerial parts of the plant	GC-MS	a-pinene, methyl geranate, b-ocimene, and limonene, a-Pinene, methyl geranate, limonene, b-ocimene, geraniol trans-verbenol, and terpinen-4-ol.	[44, 63]

25	Tehran	Whole plant	GC-MS	Flavonoids luteolin-7-O-glucoside, apigenin-7-O-glucoside (cosmosiin), luteolin 3′-O-*β*-d-glucuronide, luteolin, apigenin, cirsimaritin, isokaempferide, penduletin, xanthomicrol, calycopterin and the polyphenol rosmarinic acid	[64]

26	Isfahan	Aerial parts	GC-MS	Citral, p-mentha-1,3,8-triene, D-3-carene and methyl geranate	[65]

27	Isfahan	Whole plant	GC-MS	Apigenin	[45]

28	Tehran	Essential oil Aerial part of the plant	MTT Essay	Limonene, Geranial, 1,1-Dimethoxy decane, C10 H14O, CxHy, CxHyOz, Unidentified compound	[39]

29	Tehran	Leaf	GC-MS	Limonene, limonene-10-al, 1,1-dimethoxy decane, cyclic monoterpenes	[18]

30	Bojnord and the surrounding areas in North Khorasan	Essential oil Aerial parts	GC-MS	Trans-citral (geranial), eucalyptol, limonene, beta-linalool, neryl-acetate and myrcene.	[17]

31	Isfahan	Powder (Aerial part)	GC-MS	Luteolin, Naringenin, Apigenin, kaempferoide, Cirsimaritin, Penduletin Xanthomicrol, Calycoperin	[49]

32	Northeastern Iran	Leaf	MTT Essay	Citral, caryophyllene, terpinyl acetate, and myrcene	[24]

33	Tehran	Powder (Seeds and leaf)	GC-MS	Xanthomicrol ((5,4′-dihydroxy-6,7,8-trimethoxyflavone)	[66]

34	Isfahan	Powder (Aerial part)	GC-MS	Calycopterin	[31]

35	West Azerbaijan	Whole plant (extract)	GC-MS	(E)-*β*-ocimene, nerol, nerol/methyl, geranate, and *α*-pinene/geranial/ geraniol	[14]

36	Isfaham	Essential oil (Aerial parts)	GC-MS	(flavonoids) luteolin-7-O-glucoside, apigenin-7-O-glucoside (cosmosiin), luteolin 3′-O-*β*-d-glucuronide, luteolin, apigenin, cirsimaritin, isokaempferide, penduletin, xanthomicrol, calycopterin and the polyphenol rosmarinic acid	[66]

37	Tehran		GC-MS	Arosmarinic acid, flavonoids Limonene, neral, geranial, geraniol, geranyl acetate, *α*-terpineol, trans-verbenol, carvon, and transcarveol	[62, 63]

38	Azerbaijan	Whole plant (Powder)	GC-MS	Limonene, neral, geranial, *β*-sitosteral, Oleanolic acid, Ursolic acic, *p*-metha-8-en-1,2-diol, colosonic acid, limonen-10-ol-*O-β*-_D-_glucopyranoside, limonen-10-ol-*O-β*-_D-_glucopyranosyl-(1→2)- *β*-_D-_glucopyranoside	[11]

Note: GC-MS: gas chromatography-mass spectrometry; MTT: Ministerio de Transportes y Telecomunicacione; HPLC: high-performance liquid chromatography; NMR: nuclear magnetic resonance.

## Data Availability

The original research articles included in this systematic review are publicly available.

## Abbreviations

*D. kotschyi*: * Dracocephalum kotschyi*
PRISMA: Preferred Reporting Items for Systematic Reviews and Meta-Analyses
CDC: Center for Disease Control and Prevention.

## References

[1] Hooper D., Field H. (1937). *Useful Plants and Drugs of Iran and Iraq*.

[2] Kinghorn A. D., Pan L., Fletcher J. N., Chai H. (2011). The relevance of higher plants in lead compound discovery programs. *Journal of Natural Products*.

[3] Newman D. J., Cragg G. M. (2012). Natural products as sources of new drugs over the 30 years from 1981 to 2010. *Journal of Natural Products*.

[4] Dobrek Ł., Thor P. J. (2012). Bladder urotoxicity pathophysiology induced by the oxazaphosphorine alkylating agents and its chemoprevention. *Postepy Higieny i Medycyny Doswiadczalnej*.

[5] Lazarević P., Lazarević M., Krivošej Z., Stevanović V. (2009). On the distribution of *Dracocephalum ruyschiana* (*Lamiaceae*) in the Balkan Peninsula. *Phytologia Balcanica*.

[6] Sonboli A., Gholipour A., Mirjalili M. H., Rad M. A. (2011). Molecular characterization of Iranian *Dracocephalum* (Lamiaceae) species based on RAPD data. *Acta Biologica Szegediensis*.

[7] Mozaffarian V. (2008). *A Dictionary of Iranian Plant Names: Latin, English, Persian*.

[8] Ashrafi B., Ramak P., Ezatpour B., Talei G. R. (2017). Investigation on chemical composition, antimicrobial, antioxidant, and cytotoxic properties of essential oil from *Dracocephalum kotschyi* Boiss. *African Journal of Traditional, Complementary and Alternative Medicines*.

[9] Sadraei H., Asghari G., Alinejad M. (2016). Comparison of antispasmodic effect of hydroalcoholic extract of *Dracocephalum kotschyi* Boiss. in rat uterus and ileum. *Research in Pharmaceutical Sciences*.

[10] Gohari A. R., Saeidnia S., Matsuo K. (2003). Flavonoid constituents of *Dracocephalum kotschyi* growing in iran and their trypanocidal activity. *Journal of Natural Medicines*.

[11] Saeidnia S., Gohari A. R., Uchiyama N., Ito M., Honda G., Kiuchi F. (2004). Two new monoterpene glycosides and trypanocidal terpenoids from *Dracocephalum kotschyi*. *Chemical & Pharmaceutical Bulletin*.

[12] Saeidnia S., Gohari A. R., Ito M., Kiuchi F., Honda G. (2005). Bioactive constituents from *Dracocephalum subcapitatum* (O. Kuntze) lipsky. *Zeitschrift fur Naturforschung - Section C Journal of Biosciences*.

[13] Zeng Q., Jin H. Z., Qin J. J. (2010). Chemical constituents of plants from the genus *Dracocephalum*. *Chemistry & Biodiversity*.

[14] Fattahi M., Nazeri V., Torras-Claveria L. (2013). Identification and quantification of leaf surface flavonoids in wild-growing populations of *Dracocephalum kotschyi* by LC-DAD-ESI-MS. *Food Chemistry*.

[15] Jalaei Z., Fattahi M., Aramideh S. (2015). Allelopathic and insecticidal activities of essential oil of *Dracocephalum kotschyi* Boiss. from Iran: a new chemotype with highest limonene-10-al and limonene. *Industrial Crops and Products*.

[16] Middleton E., Kandaswami C., Theoharides T. C. (2000). The effects of plant flavonoids on mammalian cells: implications for inflammation, heart disease, and cancer. *Pharmacological Reviews*.

[17] Babbar N., Oberoi H. S., Uppal D. S., Patil R. T. (2011). Total phenolic content and antioxidant capacity of extracts obtained from six important fruit residues. *Food Research International*.

[18] Talari M., Seydi E., Salimi A., Mohsenifar Z., Kamalinejad M., Pourahmad J. (2014). *Dracocephalum*: novel anticancer plant acting on liver cancer cell mitochondria. *BioMed Research International*.

[19] Kamali M., Khosroyar S., Mohammadi A. (2015). Antibacterial activity of various extracts from *Dracocephalum kotschyi* against food pathogenic microorganisms. *International Journal of PharmTech Research*.

[20] Mashak B., Hoseinzadeh M., Ehsanpour A., Ghanbaran A. R., Vakili M. (2017). Evaluation of treatment response and side effects of spinal-Z in patients with metastatic gastroesophageal adenocarcinoma: a double-blind randomized controlled trial. *Jundishapur Journal of Chronic Disease Care*.

[21] Sajjadi S. E., Atar A. M., Yektaian A. (1998). Antihyperlipidemic effect of hydroalcoholic extract, and polyphenolic fraction from *Dracocephalum kotschyi* Boiss. *Pharmaceutica Acta Helvetiae*.

[22] Amirghofran Z., Azadbakht M., Karimi M. H. (2000). Evaluation of the immunomodulatory effects of five herbal plants. *Journal of Ethnopharmacology*.

[23] Du Toit K., Buthelezi S., Bodenstein J. (2009). Anti-inflammatory and antibacterial profiles of selected compounds found in South African propolis. *South African Journal of Science*.

[24] Fattahi M., Nazeri V., Torras-Claveria L. (2013). A new biotechnological source of rosmarinic acid and surface flavonoids: hairy root cultures of *Dracocephalum kotschyi* Boiss. *Industrial Crops and Products*.

[25] Golshani S., Karamkhani F., Monsef-Esfehani H. R., Abdollahi M. (2004). Antinociceptive effects of the essential oil of *Dracocephalum kotschyi* in the mouse writhing test. *Journal of Pharmacy & Pharmaceutical Sciences*.

[26] Jahaniani F., Ebrahimi S. A., Rahbar-Roshandel N., Mahmoudian M. (2005). Xanthomicrol is the main cytotoxic component of *Dracocephalum kotschyii* and a potential anti-cancer agent. *Phytochemistry*.

[27] Moghaddam G., Ebrahimi S. A., Rahbar-Roshandel N., Foroumadi A. (2012). Antiproliferative activity of flavonoids: influence of the sequential methoxylation state of the flavonoid structure. *Phytotherapy Research*.

[28] Sani T. A., Mohammadpour E., Mohammadi A. (2017). Cytotoxic and apoptogenic properties of *Dracocephalum kotschyi* aerial part different fractions on calu-6 and mehr-80 lung cancer cell lines. *Farmacia*.

[29] Sultan A., Bahang, Aisa H. A., Eshbakova K. A. (2008). Flavonoids from *Dracocephalum moldavica*. *Chemistry of Natural Compounds*.

[30] Ashokkumar P., Sudhandiran G. (2008). Protective role of luteolin on the status of lipid peroxidation and antioxidant defense against azoxymethane-induced experimental colon carcinogenesis. *Biomedicine & Pharmacotherapy*.

[31] Sarvestani N. N., Khodagholi F., Ansari N., Farimani M. M. (2013). Involvement of p-CREB and phase II detoxifying enzyme system in neuroprotection mediated by the flavonoid calycopterin isolated from *Dracocephalum kotschyi*. *Phytomedicine*.

[32] Farimani M., Sarvestani N., Ansari N., Khodagholi F. (2011). Calycopterin promotes survival and outgrowth of neuron-like PC12 cells by attenuation of oxidative- and ER-stress-induced apoptosis along with inflammatory response. *Chemical Research in Toxicology*.

[33] Park C. M., Jin K.-S., Cho C. W. (2012). Luteolin inhibits inflammatory responses by downregulating the JNK, NF-*κ*B, and AP-1 pathways in TNF-*α* activated HepG2 cells. *Food Science and Biotechnology*.

[34] Moghaddam G., Ebrahimi S. A., Rahbar-Roshandel N., Foroumadi A. (2012). Antiproliferative activity of flavonoids influence of the sequential Phytochemical screening of *Dracocephalum kotschyi*. *Iranian Journal of Pharmaceutical Research*.

[35] Inouye S., Takizawa T., Yamaguchi H. (2001). Antibacterial activity of essential oils and their major constituents against respiratory tract pathogens by gaseous contact. *Journal of Antimicrobial Chemotherapy*.

[36] Chachoyan A. A., Oganesyan G. B. (1996). Antitumor of some species of family Lamiaceae. *Rastitel'nye Resursy*.

[37] Kakasy A. Z., Lemberkovics É., Simándi B. (2006). Comparative study of traditional essential oil and supercritical fluid extracts of Moldavian dragonhead (*Dracocephalum moldavica* L.). *Flavour and Fragrance Journal*.

[38] Dastmalchi K., Damien Dorman H. J., Koşar M., Hiltunen R. (2007). Chemical composition and in vitro antioxidant evaluation of a water-soluble Moldavian balm (*Dracocephalum moldavica* L.) extract. *LWT- Food Science and Technology*.

[39] Aqel M., Hadidi M. (2008). Direct relaxant effect of *Peganum Harmala* seed extract on smooth muscles of rabbit and Guinea pig. *International Journal of Pharmacognosy*.

[40] Sadraei H., Ghanadian M., Asghari G., Azali N. (2014). Antidiarrheal activities of isovanillin, iso-acetovanillon and Pycnocycla spinosa Decne ex.Boiss extract in mice. *Research in Pharmaceutical Sciences*.

[41] Moher D., Liberati A., Tetzlaff J., Altman D. G. (2009). Preferred reporting items for systematic reviews and meta-analyses: the PRISMA statement. *PLoS Medicine*.

[42] Rechinger K. H., Hedge I. C., Ietswaart J. H., Jalas J., Mennema J., Seybold S., Rechinger K. H. (1982). Labiatae. *Flora Iranica*.

[43] Mashayekhan A., Pourmajidian M. R., Jalilvand H., Gholami M. R., Teimouri M. S. (2015). Ethno botanical survey of herbal remedies traditionally used in North Khorasan Province of Iran. *Medicinal & Aromatic Plants*.

[44] Agarwal S. G., Kapahi B. K., Thappa R. K. (2005). Essential oil constituents of Himalayan *Dracocephalum speciosum* benth. *Journal of Essential Oil Research*.

[45] Rechinger K. H. (1986). Cousinia: morphology, taxonomy, distribution and phytogeographical implications. *Proceedings of the Royal Society of Edinburgh. Section B. Biological Sciences*.

[46] Ghorbani A., Kavianpoor H., Sharifi-Rad M., Sharifi-Rad J. (2014). Biodiversity, life forms and chorotypes of threatend medicinal plants in Tehran watershed, Iran. *Research in Plant Biology*.

[47] Jalali A., Jamzad Z. (1999). *Red Data Book of Iran*.

[48] Lee S. B., Cha K. H., Kim S. N. (2007). The antimicrobial activity of essential oil from *Dracocephalum foetidum* against pathogenic microorganisms. *Journal of Microbiology*.

[49] Sadraei H., Asghari G., Khanabadi M., Minaiyan M. (2017). Anti-inflammatory effect of apigenin and hydroalcoholic extract of *Dracocephalum kotschyi* on acetic acid-induced colitis in rats. *Research in Pharmaceutical Sciences*.

[50] Javidnia K., Miri R., Kamalinejad M., Khoshneviszadeh M. (2006). Constituents of the volatile oils of *Dracocephalum kotschyi* Boiss. from Iran. *Journal of Essential Oil Research*.

[51] Yaghmai M. S., Taffazoli R. (1988). The essential oil of *Dracocephalum kotschyi* Boiss. *Flavour and Fragrance Journal*.

[52] Golparvar A. R., Hadipanah A., Gheisari M. M., Khaliliazar R. (2016). Chemical constituents of essential oil of Dracocephalum moldavica L. and *Dracocephalum kotschyi* Boiss. from Iran. *Acta Agriculturae Slovenica*.

[53] Goli S. A. H., Sahafi S. M., Rashidi B., Rahimmalek M. (2013). Novel oilseed of *Dracocephalum kotschyi* with high n-3 to n-6 polyunsaturated fatty acid ratio. *Industrial Crops and Products*.

[54] Monsef-Esfahani H. R., Karamkhani F., Nickavar B., Abdi K., Faramarzi M. A. (2007). The volatile constituents of *Dracocephalum kotschyi* oils. *Chemistry of Natural Compounds*.

[55] Alesheikha P., Feyzia P., Kamalib M., Sania T. A. (2016). Evaluation of antioxidant activity of Luteolin, isolated from *Dracocephalum kotschyi*. *Journal of Medicinal Plants & Natural Products*.

[56] Morteza-Semnani K., Saeedi M. (2005). Essential oil composition of *Dracocephalum kotschyi* Boiss. *Journal of Essential Oil Bearing Plants*.

[57] Sonboli A., Mirzania F., Gholipour A. (2018). Essential oil composition of *Dracocephalum kotschyi* Boiss. from Iran. *Natural Product Research*.

[58] Asghari G., Keyhanfard N. (2014). Seasonal variation of mono- and sesquiterpenoid components in the essential oil of *Dracocephalum kotschyi* Boiss. *Research Journal of Pharmacognosy*.

[59] Asghari G., Akbari M., Asadi-Samani M. (2017). Phytochemical analysis of some plants from Lamiaceae family frequently used in folk medicine in Aligudarz region of Lorestan Province. *Marmara Pharmaceutical Journal*.

[60] Badparva E., Badparva S., Mousav S. F., Mahmoudv H. (2017). In vitro effect of some Iranian medicinal plants on the *Histomonas meleagridis* parasite. *International Journal of Pharmaceutical Sciences and Research*.

[61] Ebrahimi S. A., Jahaniani Kenari F. (2013). Interaction between cytotoxic effects of Xanthomicrol and Noscapine on PC12 cells. *The Journal of Genes, Microbes and Immunity*.

[62] Eskandari A., Heidari R., Farokhi F., Salimi Z., Ghasemi Z. (2012). Effect of aqueous extract from rhizome of Cynodon dactylon Lpers on renal and hepatic catalase activity and testicular histopathology in diabetic. *Feyz Journal of Kashan University of Medical Sciences*.

[63] Faghihinia L., Monajjemi R., Ranjbar M. (2015). Cytotoxic and antioxidant effects of methanol, hexane, chloroform and aqueous extracts of *Dracocephalum kotschyi* aerial parts on MDA-MB-231 cell line. *Journal of Biodiversity and Environmental Sciences*.

[64] Hassani M., Farahpour M., Mahdavi M., Hassani L. (2013). Study of essantial oil content and composition of *Dracocephalum Kotschyi* in different stages of plant growth in Mazandaran province. *Advances in Environmental Biology*.

[65] Kamali M., Khosroyar S., Kamali H., Sani T. A., Mohammadi A. (2016). Phytochemical screening and evaluation of antioxidant activities of *Dracocephalum kotschyi* and determination of its luteolin content. *Avicenna Journal of Phytomedicine*.

[66] Motlagh H. R. M., Mansouri K., Shakiba Y. (2009). Anti-angiogenic effect of aqueous extract of shallot (*Allium ascalonicum*) bulbs in rat aorta ring model. *Yakhteh Medical Journal*.

[67] Zamani S.-S., Hossieni M., Etebari M., Salehian P., Ebrahimi S. A. (2016). Pharmacokinetics of calycopterin and xanthmicrol, two polymethoxylated hydroxyflavones with anti-angiogenic activities from *Dracocephalum kotschyi* Bioss. *DARU Journal of Pharmaceutical Sciences*.

[68] Nejad-Sadeghi M., Taji S., Goodarznia I. (2015). Optimization of supercritical carbon dioxide extraction of essential oil from *Dracocephalum kotschyi* Boiss: an endangered medicinal plant in Iran. *Journal of Chromatography A*.

[69] Kalantar K., Gholijani N., Mousaei N., Yazdani M., Amirghofran Z. (2018). Investigation of *Dracocephalum kotschyi* plant extract on the effective inflammatory transcription factors and mediators in activated macrophages. *Anti-Inflammatory & Anti-Allergy Agents in Medicinal Chemistry*.

[70] Faham N., Javidnia K., Bahmani M., Amirghofran Z. (2008). Calycopterin, an immunoinhibitory compound from the extract of *Dracocephalum kotschyi*. *Phytotherapy Research*.

[71] Moridi Farimani M., Mirzania F., Sonboli A., Moghaddam F. M. (2017). Chemical composition and antibacterial activity of *Dracocephalum kotschyi* essential oil obtained by microwave extraction and hydrodistillation. *International Journal of Food Properties*.

[72] Sadraei H., Asghari G., Kasiri F. (2015). Comparison of antispasmodic effects of *Dracocephalum kotschyi* essential oil, limonene and *α*-terpineol. *Research in Pharmaceutical Sciences*.

[73] Esmaeili M. A., Farimani M. M., Kiaei M. (2014). Anticancer effect of calycopterin via PI3K/Akt and MAPK signaling pathways, ROS-mediated pathway and mitochondrial dysfunction in hepatoblastoma cancer (HepG2) cells. *Molecular and Cellular Biochemistry*.

[74] Graidist P., Martla M., Sukpondma Y. (2015). Cytotoxic activity of *Piper cubeba* extract in breast cancer cell lines. *Nutrients*.

[75] Zee-Cheng R. K. (1997). Anticancer research on Loranthaceae plants. *Drugs of the Future*.

[76] Takahashi T., Kobori M., Shinmoto H., Tsushida T. (1998). Structure-activity relationships of flavonoids and the induction of granulocytic- or monocytic-differentiation in HL60 human myeloid leukemia cells. *Bioscience, Biotechnology, and Biochemistry*.

[77] Wu B., Zhang Q., Shen W., Zhu J. (2008). Anti-proliferative and chemosensitizing effects of luteolin on human gastric cancer AGS cell line. *Molecular and Cellular Biochemistry*.

[78] Manthey J. A., Guthrie N. (2002). Antiproliferative activities of citrus flavonoids against six human cancer cell lines. *Journal of Agricultural and Food Chemistry*.

[79] Kawaii S., Tomono Y., Katase E., Ogawa K., Yano M. (1999). Antiproliferative activity of flavonoids on several cancer cell lines. *Bioscience, Biotechnology, and Biochemistry*.

[80] Martin T. A., Ye L., Sanders A. J., Lane J., Jiang W. G., Jandial R. (2013). Cancer invasion and metastasis: molecular and cellular perspective. Metastatic cancer: clinical and biological perspectives. *Madame Curie Bioscience Database*.

[81] Hassan Z. M., Feyzi R., Sheikhian A. (2005). Low molecular weight fraction of shark cartilage can modulate immune responses and abolish angiogenesis. *International Immunopharmacology*.

[82] Shakiba Y., Mansouri K., Mostafaie A. (2007). Anti-angiogenic effect of soybean kunitz trypsin inhibitor on human umbilical vein endothelial cells. *Fitoterapia*.

[83] D’Andrea L., Del Gatto A., Pedone C., Benedetti E. (2006). Peptide-based molecules in angiogenesis. *Chemical Biology & Drug Design*.

[84] Rahimi N. (2006). Vascular endothelial growth factor receptors: molecular mechanisms of activation and therapeutic potentials. *Experimental Eye Research*.

[85] Amirifard N., Sadeghi E., Payandeh M., Mohebbi H., Sadeghi M., Choubsaz M. (2016). Relationship between HER2 proto-oncogene status and prognostic factors of breast cancer in the West of Iran. *Asian Pacific Journal of Cancer Prevention*.

[86] Mukhtar E., Adhami V. M., Khan N., Mukhtar H. (2012). Apoptosis and autophagy induction as mechanism of cancer prevention by naturally occurring dietary agents. *Current Drug Targets*.

[87] Cardone M. H., Roy N., Stennicke H. R. (1998). Regulation of cell death protease caspase-9 by phosphorylation. *Science*.

[88] Lee J., Jo D.-G., Park D., Chung H. Y., Mattson M. P. (2014). Adaptive cellular stress pathways as therapeutic targets of dietary phytochemicals: focus on the nervous system. *Pharmacological Reviews*.

[89] Karrer W., Venkataraman K. (1935). Identity of calycopterin and thapsin. *Nature*.

[90] Green S. J., Mellouk S., Hoffman S. L., Meltzer M. S., Nacy C. A. (1990). Cellular mechanisms of nonspecific immunity to intracellular infection: cytokine-induced synthesis of toxic nitrogen oxides from L-arginine by macrophages and hepatocytes. *Immunology Letters*.

[91] Bosani M., Ardizzone S., Porro G. B. (2009). Biologic targeting in the treatment of inflammatory bowel diseases. *Biologics: Targets & Therapy*.

[92] Kalantar F., Hosseini S. M., Gholmoghadam H., Shabani M., Amirghofran Z. (2013). Inhibition of nitric oxide production and proinflammatory cytokines by several medicinal plants. *Frontiers in Immunology*.

[93] Janbaz K. H., Saeed S. A., Gilani A. H. (2002). Protective effect of rutin on paracetamol- and CCl4-induced hepatotoxicity in rodents. *Fitoterapia*.

[94] López-Lázaro M. (2009). Distribution and biological activities of the flavonoid luteolin. *Mini-Reviews in Medicinal Chemistry*.

[95] Shakib P., Taherikalani M., Ramazanzadeh R. (2018). Chemical composition, genotoxicity and antimicrobial activities of *Dracocephalum kotschyi* Boiss against OXA-48 producing *Klebsiella pneumoniae* Isolated from major hospitals of Kurdistan Province, Iran. *Microbiology Research Journal International*.

[96] Manel K., Hanen F., Kamel M. (2016). Antioxidant, Anti-inflammatory and anticancer activities of the medicinal halophyte Reaumuria vermiculata. *EXCLI Journal*.

[97] Seth R., Mohan M., Singh P. (2012). Chemical composition and antibacterial properties of the essential oil and extracts of *Lantana camara* Linn. from Uttarakhand (India). *Asian Pacific Journal of Tropical Biomedicine*.

[98] Dorman H. J. D., Deans S. G. (2000). Antimicrobial agents from plants: antibacterial activity of plant volatile oils. *Journal of Applied Microbiology*.

[99] Duarte M. C., Leme E. E., Delarmelina C., Soares A. A., Figueira G. M., Sartoratto A. (2007). Activity of essential oils from Brazilian medicinal plants on *Escherichia coli*. *Journal of Ethnopharmacology*.

[100] Singh S. K., Vishnoi R., Dhingra G. K., Kishor K. (2012). Antibacterial activity of leaf extracts of some selected traditional medicinal plants of Uttarakhand, North East India. *Journal of Applied and Natural Science*.

[101] Maksimović Z., Stojanović D., Šoštarić I., Dajić Z., Ristić M. (2008). Composition and radical-scavenging activity of *Thymus glabrescens* Willd. (Lamiaceae) essential oil. *Journal of the Science of Food and Agriculture*.

[102] Sartoratto A., Machado A. L. M., Delarmelina C., Figueira G. M., Duarte M. C. T., Rehder V. L. G. (2004). Composition and antimicrobial activity of essential oils from aromatic plants used in Brazil. *Brazilian Journal of Microbiology*.

[103] Aguiar G. P., Melo N. I., Wakabayashi K. A. L. (2013). Chemical composition and *in vitro* schistosomicidal activity of the essential oil from the flowers of *Bidens sulphurea* (Asteraceae). *Natural Product Research*.

[104] Öztürk M., Duru M. E., Aydoğrmuş-Öztürk F. (2009). GC-MS analysis and antimicrobial activity of essential oil of *Stachys Cretica* Subsp. *Smyrnaea*. *Natural Product Communications (NPC)*.

[105] Ballester-Costa C., Sendra E., Fernández-López J., Pérez-Álvarez J. A., Viuda-Martos M. (2013). Chemical composition and in vitro antibacterial properties of essential oils of four *Thymus* species from organic growth. *Industrial Crops and Products*.

[106] Orlanda J. F., Nascimento A. R. (2015). Chemical composition and antibacterial activity of *Ruta graveolens* L. (Rutaceae) volatile oils, from São Luís, Maranhão, Brazil. *South African Journal of Botany*.

[107] Shakeri A., Khakdan F., Soheili V., Sahebkar A., Rassam G., Asili J. (2014). Chemical composition, antibacterial activity, and cytotoxicity of essential oil from *Nepeta ucrainica* L. spp. kopetdaghensis. *Industrial Crops and Products*.

[108] Nikaido H., Vaara M. (1985). Molecular basis of bacterial outer membrane permeability. *Microbiology and Molecular Biology Reviews*.

[109] Wendakoon C. N., Sakaguchi M. (1993). Combined effect of sodium chloride and clove on growth and biogenic amine formation of *Enterobacter aerogenes* in mackerel muscle extract. *Journal of Food Protection*.

[110] Okach D. O., Nyunja A. R. O., Opande G. (2013). Phytochemical screening of some wild plants from Lamiaceae and their role in traditional medicine in Uriri District-Kenya. *International Journal of Herbal Medicine*.

[111] Zargari A. (1990). *Medicinal Plants*.

[112] Zargari A. (2000). *Medicinal Plants*.

[113] Grayer R. J., Veitch N. C., Kite G. C., Price A. M., Kokubun T. (2001). Distribution of 8-oxygenated leaf-surface flavones in the genus *Ocimum*. *Phytochemistry*.

[114] Lee S. M. Y., Li M. L. Y., Tse Y. C. (2002). *Paeoniae Radix*, a Chinese herbal extract, inhibit hepatoma cells growth by inducing apoptosis in a p53 independent pathway. *Life Sciences*.

[115] Song Y., Kesuma D., Wang J. (2004). Specific inhibition of cyclin-dependent kinases and cell proliferation by harmine. *Biochemical and Biophysical Research Communications*.

[116] Zargari A. (1995). *Medicinal Plants*.

