# Corrigendum to “Medicinal Properties and Active Constituents of* Dracocephalum kotschyi* and Its Significance in Iran: A Systematic Review”

**DOI:** 10.1155/2019/5607329

**Published:** 2019-07-21

**Authors:** Parisa Heydari, Maryam Yavari, Peyman Adibi, Gholamreza Asghari, Syed-Mustafa Ghanadian, Gabriel O. Dida, Faham Khamesipour

**Affiliations:** ^1^Department of Persian Medicine, Faculty of Medicine, Isfahan University of Medical Sciences, Isfahan, Iran; ^2^Department of Traditional Medicine, Isfahan University of Medical Sciences, Isfahan, Iran; ^3^Integrative Functional Gastroenterology Research Center, Isfahan University of Medical Sciences, Isfahan, Iran; ^4^Department of Pharmacognosy, School of Pharmacy and Pharmaceutical Sciences, Isfahan University of Medical Sciences, Isfahan, Iran; ^5^Department of Pharmacognosy, Faculty of Pharmacy, Isfahan University of Medical Sciences, Isfahan, Iran; ^6^School of Public Health and Community Development, Maseno University, Kenya; ^7^Department of Community and Public Health, Technical University of Kenya, Nairobi, Kenya; ^8^Cellular and Molecular Research Center, Sabzevar University of Medical Sciences, Sabzevar, Iran; ^9^Shahid Beheshti University of Medical Sciences, Tehran, Iran

In the article titled “Medicinal Properties and Active Constituents of Dracocephalum kotschyi and Its Significance in Iran: A Systematic Review” [1], Dr. Peyman Adibi should be listed as the second corresponding author.
